# The 5Ms of Geriatrics in Gastroenterology: The Path to Creating Age-Friendly Care for Older Adults With Inflammatory Bowel Diseases and Cirrhosis

**DOI:** 10.14309/ctg.0000000000000445

**Published:** 2022-01-12

**Authors:** Bharati Kochar, Nneka N. Ufere, Christine S. Ritchie, Jennifer C. Lai

**Affiliations:** 1 Division of Gastroenterology, Massachusetts General Hospital, Boston, Massachusetts, USA;; 2 Harvard Medical School, Boston, Massachusetts, USA;; 3 The Mongan Institute, Boston, Massachusetts, USA;; 4 Division of Palliative Care and Geriatric Medicine, Massachusetts General Hospital, Boston, Massachusetts, USA;; 5 Division of Gastroenterology and Hepatology, University of California San Francisco, San Francisco, California, USA.

## Abstract

The number of Americans 65 years or older in 2060 will be more than double what it was in 2014. Approximately 40% of patients seen in gastroenterology (GI) and hepatology practices in the United States are 60 years or older. Adapting care delivery models, curating data on shifting risk-benefit decisions with geriatric syndromes, understanding appropriate assessments, and focusing on tailored implementation strategies are challenges that are actively confronting us as we provide care for a burgeoning population of older adults. Limited availability of geriatric specialists results in an onus of specialists caring for older adults, such as gastroenterologists, to innovate and develop tailored, comprehensive, and evidence-based care for adults in later life stages. In this article, we present the 5M framework from geriatrics to achieve age-friendly healthcare. The 5Ms are medications, mind, mobility, multicomplexity, and what matters most. We apply the 5M framework to 2 chronic conditions commonly encountered in clinical GI practice: inflammatory bowel diseases and cirrhosis. We highlight knowledge gaps and outline future directions to expand evidence-based care and advance the creation of age-friendly GI care.

## INTRODUCTION

The 2020 US Census reported 73 million Americans were 65 years or older, a 34% increase from the previous decade, making older adults the fastest growing age group in the United States (1). By 2034, older adults are projected to outnumber children for the first time in the history of the United States (2). Concomitantly, life expectancy has increased 30 years in the past 100 years, the biggest change in the history of humanity (3). While increasing lifespan used to be the Holy Grail, arguably more important, is increasing health span—the duration of time people can remain healthy (4).

The challenges of providing care to a rapidly aging population are readily apparent in the routine clinical practice of gastroenterology (GI), where at least 40% of patients are 60 years or older (5). Approximately 60% of new diagnoses of colorectal cancer are in patients aged 65 years or older and nearly 20% of cases are in patients aged 80 years or older (6). The physiological effects of aging contribute to the high prevalence of common GI conditions such as constipation (16%–26%), malnutrition (5%–40%), and gastroesophageal reflux disease (6%–17%) among older adults (7–9). Older adults are also more susceptible to hepatotoxicity and drug-induced liver injury (10). GI clinicians face a number of conundrums in caring for older adults: such as timing of colon cancer screening cessation, higher endoscopy complication rates, lower thresholds for liver injury, and age-appropriate GI symptom management. However, there is a large data void to guide clinical decisions in caring for older adults in GI practice. Because older adults increase in number and live longer, being equipped to address the dilemmas and poised to provide comprehensive care for our aging patients will be one of the greatest challenges to our profession.

In addition to the high prevalence of common GI symptoms among older adults, there is also an increasing prevalence of older adults with chronic GI conditions such as inflammatory bowel diseases (IBDs) and cirrhosis. Although IBD and cirrhosis are not traditionally considered geriatric conditions, the burden of these conditions in older adults in the United States is rapidly increasing. A 2015 report by the Centers for Disease Control estimated that 26% of Americans living with IBD are 65 years or older (11). A modeling study projected that in 2030, the number of adults aged 65 years or older with IBD will be >200% what it was in 2008 (12). Similarly, the proportion of cirrhotic patients aged 65 years or older increased from 24% to 33% between 2000 and 2014 (13–15). More strikingly, between 2002 and 2014, the proportion of patients aged 60 years or older on the liver transplantation waiting list increased from 19% to 41% (14). Aging-related morbidities and impairments may complicate traditional guideline-based management of IBD and cirrhosis. In turn, there is an emerging need for gastroenterologists to adapt their clinical armamentarium to incorporate the cognitive and functional status, comorbidities, life expectancy, and preferences of older adults into decision-making. In this narrative review, we will highlight the need to integrate geriatric constructs into the management of older adults with IBD and cirrhosis to optimize care for these complex and vulnerable patients.

## AGE-FRIENDLY HEALTHCARE

The concept in modern allopathic medicine that older adults warrant different care only emerged in the late 1880s, and the first fellowship in geriatric medicine was not established until 1966 (16). However, there are not enough trained geriatricians to provide comprehensive care for the rapidly aging population (17). Therefore, individual specialties have to innovate to conduct appropriate and comprehensive assessments of older adults, generate adequate safety and efficacy data for therapeutic agents, especially for older adults, and assess outcomes that are important to adults in later life stages.

**Figure 1.**
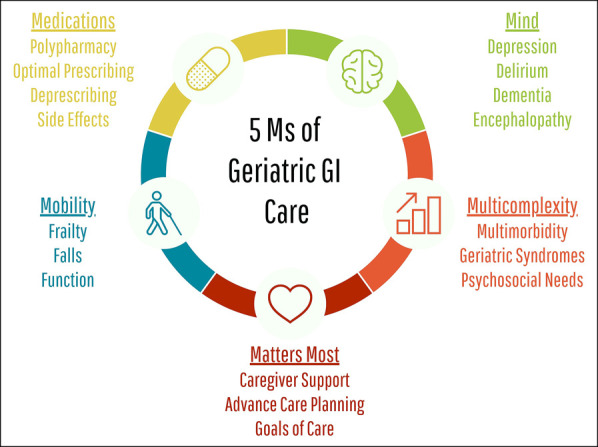
Conceptual model for providing age-friendly healthcare.

Understanding the principles of geriatrics is the first step to learning how to incorporate it into our discipline. Although the field of geriatrics is broad, the principles guiding the field are elegantly encapsulated in the 5M framework (Figure 1):

- Medications is in recognition of the rampant polypharmacy in older adults (18). Definitions of polypharmacy vary, with research studies most commonly using a numeric cutoff (≥5), but more clinically relevant measures assess appropriateness of each medication (19). It is well-accepted that appropriate medication doses and medication interactions are different in older adults, resulting in medication appropriateness lists tailored for older adults (20,21). More recently, there are important movements to deprescribe, when appropriate, to maximize the benefit to harm ratio (22).
- Mind refers to mood and memory—to prevent, identify early, treat, and manage conditions such as depression, delirium, and dementia. This acknowledges that there are many modifiable risk factors for cognitive decline in older adults (23).
- Mobility is to ensure an appropriate approach to assist and encourage older adults to move safely and maintain functional ability to perform activities of daily living and beyond (24).
- Multicomplexity is in recognition of the significant burden of multiple comorbidities, geriatric syndromes, and serious illness prevalent in older adults and to assess conditions affected by age and social concerns, such as financial vulnerability and social isolation (25).
- Matters most is a reminder to provide patient-centered care to prioritize older adults' specific health outcome goals and care preferences tailored to their overall health and life (23).

The 5Ms hold intersectionality: the relationships between and among each is critically important. This framework results in care tailored to the needs of older adults that leads to improved outcomes for the complex and vulnerable population that we increasingly see in the practice of medicine (23). Unfortunately, older adults are not adequately represented in clinical trials in GI. In IBD clinical trials, upper age limits and exclusion criteria that include medical comorbidities and cognitive impairment disproportionately affect the recruitment and enrollment of older adults (26). Therefore, data are needed to understand the optimal management of older adults are also lacking, posing a significant burden to gastroenterologists who treat older adults. To understand where our field is and how to advance to providing the best possible care for our older patients, we propose a framework to consider the 5Ms in IBD and cirrhosis, 2 examples of chronic GI conditions with increasing prevalence among older adults.

## THE 5Ms AND IBD

The aging population, decreasing fatality of IBD, and rise in incidence and prevalence result in a prominent increase in the number of older adults with IBD (27,28). Although IBD treatments have improved, years of life lived with disability has doubled over time with a peak in the seventh decade of life (28). Patients with IBD present with geriatric syndromes, such as osteoporosis, hip fractures, polypharmacy, frailty, serious infections, and malignancies at earlier ages, independent of treatment (29–35). Using the 5M framework may help better elucidate the prominent components needed to increase health span in the rapidly aging population with IBD. It will also highlight the intersectionality of these constructs that need to be better elucidated as they pertain specifically to older adults with IBD.

### Medications

The approved and available treatment options for IBD have rapidly proliferated over the past 2 decades. However, <1% of adults in trials of IBD medications approved after the year 2000 are 65 years or older (26). This may be one reason why older adults with IBD are more likely to receive corticosteroids over steroid-sparing therapies despite the known significant consequences of markedly higher risks for venous thromboembolism, fracture, and infections in older adults with IBD treated with steroids (36–38). Studies note that older adults may have differing responses and risk-benefit profiles with medical therapy compared with younger adults (39,40). Furthermore, older adults have higher baseline risks for adverse events such as malignancies and infections (41,42). For example, baseline lymphoma risks are higher in older adults; doubling the baseline risk may partially explain the increased rates of lymphoproliferative disorders noted in older adults treated with thiopurines (43). There is an urgent need to generate safety and efficacy data for agents used to treat IBD, especially for older adults. In addition, drug interactions are not well studied for newer immunosuppressive medications. Beyond anti-inflammatory treatments, patients with IBD are often treated symptomatically with antidiarrheal drugs, antiemetics, and concomitant treatment for irritable bowel syndrome. Many of the agents used for management of these symptoms, such as diphenoxylate/atropine, hyoscyamine, and tricyclic antidepressants, carry significant anticholinergic properties, which may compound with other medications to cause significant anticholinergic side effects in older adults. Progressive and cumulative anticholinergic burden can also lead to falls and cognitive decline in older adults (44). A detailed review of medications and their indications at each visit is warranted. Furthermore, deprescribing practices are not well studied in IBD. Understanding if and when to de-escalate longitudinal thiopurine therapy or mesalamine therapy are questions often raised in clinic.

### Mind

Although the gut–brain axis is well-described, it is poorly understood. Patients with IBD experience high rates of depression and other mood disorders (45). While many IBD clinics screen patients for depression, older adults manifest depression differently than younger adults. Therefore, screening for mood disorders using tools validated in younger adults may not detect mood disorders in older adults with high sensitivity (46). With increasing number of older adults in our clinical practice, we should study whether using a Geriatric Depression Scale will better identify older adults at risk for depression and potentially linked, worsening IBD-related outcomes (47). Detecting depression may be especially important for older adults because geriatric depression is associated with cognitive decline (48). In addition, a potential relationship between IBD and cognitive decline has been raised recently. Two retrospective studies using administrative claim databases revealed that adults with IBD are at increased risk for Parkinson disease and dementia longitudinally (49,50). These hypothesis-generating studies highlight the importance of better characterizing the gut–brain axis, especially as it pertains to older adults with IBD.

### Mobility

There are no dedicated studies of mobility in patients with IBD. However, symptoms of IBD, compounded by associated inflammatory arthralgia and/or arthritis, often render older adults homebound. The resulting social isolation may have more profound and longitudinal effects on older adults than those on younger adults. Older adults with IBD may be at increased risk for falls and fractures due to a number of factors, such as chronic steroid use resulting in osteoporosis, anticholinergic drug burden, and needing to urgently locate a restroom (30). Given age-related declines in mobility, assessing the influence of disease and drug on mobility and determining reversible causes is especially important in caring for older adults with IBD. Frailty, a concept that is interrelated with mobility, is better characterized in IBD, shown to be prevalent and associated with adverse outcomes (51).

### Multicomplexity

Older adults with IBD are at increased risk for serious infections and malignancies (33–35). Older adults with IBD are also more likely than younger adults to have serious comorbid conditions that may limit treatment strategies and increase the risk of medication side effects, such as the effect of antitumor necrosis factor agents or corticosteroids on patients with heart failure (52). Furthermore, older adults may have competing morbidities and social complexities that preclude guideline-driven treatment strategies. In addition, many guideline-based treatment recommendations may be logistically difficult to implement because of insurance constraints or lack of social support. The need to provide age-friendly interdisciplinary care, in conjunction with other specialists, such as geriatricians, cardiologists, nephrologists, and neurologists, is heightened when treating an older adult with IBD. However, IBD practitioners are often accustomed to working in multidisciplinary settings with other providers to care for complex patients (53). Recognizing the increasing medical and social complexities that accompany advancing age, while balancing the implementation of appropriate therapeutic strategies, is critical to caring for the older adult with IBD.

### Matters most

As the prevalence of older adults with IBD rises, we need to learn more about what matters most to older adults about their IBD, IBD treatments and their expectations, and their goals. While IBD affects lifespan less (28), the symptoms associated with IBD have a profound effect on health span. Retrospective studies have suggested higher rates of colectomy in older adults with ulcerative colitis (54); it is not known whether the decision-making may be elective because of fewer concerns with stomas and a desire for faster symptomatic improvement or whether the disease is truly more refractory. Similarly, there is a paucity of data guiding risk-benefit decision associated with cessation of surveillance colonoscopies in older adults with long-standing colitis. Talking to patients and learning from their experiences will teach us to focus on what matters most to the rapidly aging population of older adults with IBD.

## THE 5Ms AND CIRRHOSIS

Improvements in cirrhosis care and expanded therapeutic options for older adults with cirrhosis are 2 leading factors explaining increased aging among patients with cirrhosis (13,14,55,56). In addition to the aging of the hepatitis C baby boomer birth cohort, there is also an increasing prevalence of non-alcoholic steatohepatitis and hepatocellular carcinoma, which disproportionately affect older adults (14). Because the population of patients with cirrhosis continues to age and therapeutic options for chronic liver diseases expand, there is a critical need to address current knowledge gaps in clinical decision-making and develop research priorities with the goal of optimizing care for older patients with cirrhosis.

### Medications

Polypharmacy is common in patients with cirrhosis, who use a median of 9 medications in the first year after their diagnosis, and is associated with poor health-related quality of life including unplanned hospital readmissions (57,58). In addition, the routine use of beta-blockers, ciprofloxacin, diuretics, and lactulose for the management of complications of cirrhosis may exacerbate geriatric syndromes such as falls, cognitive impairment, urinary incontinence, and fecal incontinence, respectively, in older adults (59,60). Clinicians should perform medication reconciliation at every clinical visit and consider collaboration with pharmacists if available. The use of a pharmacist-led intervention was shown to improve knowledge, self-management, and quality of life in patients with cirrhosis (61). To date, research on optimal prescribing and deprescribing among older adults with cirrhosis has been limited and warrants further investigation.

### Mind

More than 40% of patients with cirrhosis will experience at least 1 episode of hepatic encephalopathy, a state of cognitive impairment associated with poor health-related quality of life (62,63). The presence of hepatic encephalopathy is a poor prognostic marker in cirrhosis in older adults; while patients younger than 65 years have a median survival of 2.5 years after the development hepatic encephalopathy, those aged 65 years or older have a median survival of <1 year (64). Older adults with cirrhosis are also at increased risk for aging-related cognitive impairment due to cerebrovascular disease, neurodegenerative disorders, dementia, medication side effects, delirium, and depression. These conditions may synergize with hepatic encephalopathy to negatively affect quality of life among older adults with cirrhosis and can additionally impair medication adherence and self-care activities (65). Little is known about pathophysiology of cognitive impairment in older adults with cirrhosis and, especially, the use of comprehensive neuropsychiatric testing to distinguish hepatic encephalopathy from other forms of aging-related cognitive impairment, such as dementia and delirium (66). These data are needed to counsel older patients and their families regarding likelihood of cognitive impairment after procedural interventions or cognitive recovery in the posttransplant setting.

### Mobility

Patients with cirrhosis represent a population at high risk for functional impairment, falls, and frailty. Nearly 20% of older individuals with cirrhosis experience severe functional decline, loss of ≥2 activities of daily living, over a median of 2 years, double than that of individuals without cirrhosis (67). Falls are a particularly morbid complication of cirrhosis that are independently associated with mortality. Fall prevention strategies need to be developed for this vulnerable population (68,69). Frailty, a measure of health reserve and mediator of health outcomes, serves as an example of the importance of incorporating geriatric principles into cirrhosis care to guide clinical decision-making. The Liver Frailty Index, an objective metric that uses grip strength, chair stands, and balance testing is a validated measure that is strongly associated with risk of cirrhosis disease progression, waitlist mortality, and death independent of the model for end-stage liver disease score (70–73). Frailty is now a well-accepted and validated construct in hepatology that can easily be incorporated into routine clinical care (74). Future work could assess the use of frailty testing to quantify risks of poor outcomes after transplant and nontransplant surgeries and procedures for older adults with cirrhosis.

### Multicomplexity

Multicomplexity confers markedly high risks of poor health outcomes in older adults with cirrhosis. In one nationally representative sample of older Americans, individuals with cirrhosis were found to have 3 comorbid conditions on average, most commonly arthritis (67%), hypertension (58%), and heart disease (42%) (67). Older adults with cirrhosis also require double the healthcare services compared with age-matched individuals without cirrhosis, highlighting the synergistic effect of liver disease, multimorbidity, and geriatric syndromes (67).

In clinical care, the routine assessment for multimorbidity is critical for older adults with cirrhosis due to the risk of drug-disease interactions: for example, the use of beta-blockers for variceal bleed prophylaxis in a patient with coincident chronic obstructive pulmonary disease. Clinicians should incorporate outcomes beyond mortality that are important to older adults such as functional independence, financial burden, and quality of life in shared decision-making. Clinical trials should selectively recruit patients with specific disease dyads and triads (such as nonalcoholic steatohepatitis cirrhosis in combination with chronic kidney disease and/or cardiovascular disease) to assess the effectiveness of therapeutic interventions in older adults with cirrhosis and multimorbidity (75–77).

### Matters most

While there is an increasing trend of liver transplantation among older adults with cirrhosis, older patients have higher waitlist and posttransplant mortality (14). Furthermore, many older adults with cirrhosis will not be transplant candidates due to multimorbidity. Patients with cirrhosis often receive high intensity end-of-life care, regardless of transplant eligibility, experience late advance care planning, and have low utilization of palliative care services (78–80). Older patients with cirrhosis also require twice the amount of informal caregiving compared with those without cirrhosis (67). These data highlight the increased need for early advance care planning for older adults with cirrhosis and their families to guide shared medical decision that is centered on their values, goals, and what matters most to them in life in the face of limited life expectancy (81,82).

## CLINICAL INTEGRATION

**Figure 2.**
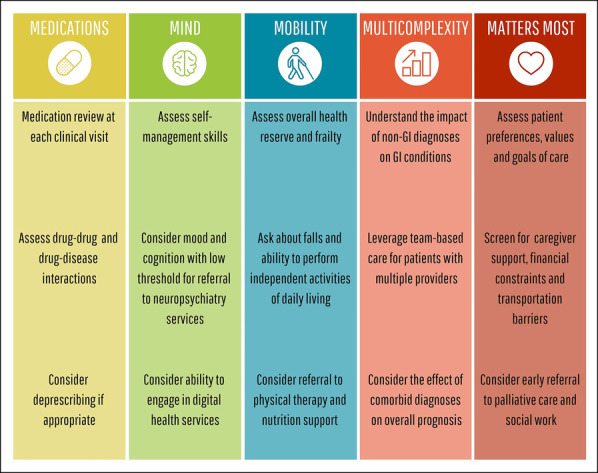
Framework for integrating principles of geriatrics into the management of older adults with chronic gastrointestinal conditions.

A number of geriatric tools and interventions are used in other specialty practices including fall screening, memory assessment, function capacity assessments, and deprescribing. However, none of these have been studied and integrated into routine GI practice. In Figure 2, we further propose a framework for integrating principles of geriatrics into the management of older adults with IBD and cirrhosis. Clinicians should be aware of the prevalence of geriatric syndromes such as frailty, cognitive and functional impairment, falls, and depression among older adults with IBD and cirrhosis and screen for these conditions where appropriate. As telemedicine and telehealth expand, clinicians should be aware of the potential effects of cognitive, visual, and auditory impairments on the ability of older adults to engage in care (5). Routine assessment of polypharmacy and multimorbidity may guide deprescribing and determining the risk-benefit ratio of future therapeutics or continued screening for colorectal cancer or hepatocellular carcinoma in these populations. Patients who are particularly complex may require comanagement through multidisciplinary and collaborative care with primary care providers and other subspecialty clinicians that may include surgery, cardiology, geriatrics, neurology, physical therapy, nutrition, palliative care, and social work, among others. Finally, GI clinicians should recognize the burden of caregiving for older adults with IBD and cirrhosis and work collaboratively with both patients and their caregivers to develop personalized approaches to care that incorporate a patient's goals, values, and preferences.

## FUTURE DIRECTIONS

There is an urgent need for evidence-based and systematic methods to guide treatment decision-making in older adults with chronic GI conditions. We summarized critical gaps in knowledge and paths for future directions to advance care for older adults with IBD and cirrhosis in Table 1. Clinical trials and registries in chronic GI conditions should be enriched with older patients and other populations with geriatric syndromes. In addition, trials and robust prospective studies should capture and assess outcomes that are relevant to older adults, including side-effect profiles, physical and cognitive function, and maintenance of independence, that is, quality of life. More data are needed to develop standardized, validated, and disease-specific metrics for polypharmacy, frailty and multimorbidity. There is also a need to develop risk assessment models that incorporate GI conditions and geriatric principles to risk stratify older patients at risk for adverse outcomes and eventually inform decision support tools. Finally, systematically developing tools and methods to guide advance care planning and shared decision-making is critical.

**Table 1.** Summary of critical gaps in knowledge and paths for future directions to advance age-friendly care for older adults with IBD and cirrhosis

	Knowledge gaps	Top research priorities
Medications	Older adults are underrepresented in IBD and cirrhosis clinical trials Limited safety and efficacy data for agents used to manage IBD and cirrhosis in older adults Lack of prescribing and deprescribing guidelines in older adults with IBD and cirrhosis	Clinical trials enriched with proportionate representation of older adults with IBD and cirrhosis to develop evidence-based and high quality treatment strategies Dedicated pharmacologic studies of medication safety and efficacy in older adults with IBD and cirrhosis Studies of time to harm versus time to benefit, including cost-effectiveness, of medications used to treat older adults with IBD and cirrhosis Qualitative research on physician, patient, and caregiver attitudes toward deprescribing for older adults with IBD and cirrhosis Randomized control trials of deprescribing for older adults with IBD and cirrhosis
Mind	Limited data on interplay among dementia, delirium, mood, and cirrhosis-related cognitive impairment Mechanisms for IBD being implicated for higher risks for mood disorders and dementia	Use of biomarkers, imaging, other modalities for assessment of neurocognitive functioning in older adults with IBD and cirrhosis Basic science and translational research characterizing the gut–brain axis in older adults with IBD and cirrhosis
Mobility	Understanding barriers to mobility in older adults with IBD and cirrhosis	Physical therapy, exercise, and dietary intervention studies to assess outcomes such as falls, frailty, functional status, patient satisfaction, and cost-effectiveness in older adults with IBD and cirrhosis Use of frailty and/or function to risk stratify older adults with IBD or cirrhosis undergoing invasive procedures
Multicomplexity	Limited generalizability of current IBD and cirrhosis clinical trials to older adults with multimorbidity and geriatric syndromes	Development of models that incorporate multimorbidity and geriatric syndromes for assessing risk and predicting clinical outcomes for older adults with IBD and cirrhosis Assessing outcomes important to older adults such as functional independence and quality of life in studies of older adults with IBD and cirrhosis
Matters most	Limited tools available to guide shared decision-making, advanced care planning, and goals of care discussions for older patients with IBD and cirrhosis	Develop and validate high quality tools to assess patient preferences for care Develop effective communication tools and skills for advance care planning and goals of care

IBD, inflammatory bowel disease.

Subspeciality organizations such as the American Society of Clinical Oncology and the American Heart Association released scientific statements in response to the critical need to improve the evidence base and clinical care for older adults with cancer and heart disease (83,84). It is time for GI organizations to pave the path to improving care for the older adults we treat as well. Future clinical guidelines from GI societies should focus on the applicability of clinical recommendations to the burgeoning population of older adults we see in practice. Collectively, the GI societies fund a substantial amount of research in the field; we recommend that they issue a request for proposals from bench to bedside focusing on aging as it pertains to GI conditions with study topics ranging from microbiome changes to shared decision making. In addition, the GI societies should create aging interest groups to advance clinical care, advocacy, and research of our older patients. Although a plethora of ideas on how to advance the field can be generated, the first big step is to acknowledge the need to adequately prepare for the rapidly growing population of older adults who need better quality care.

## CONFLICTS OF INTEREST

**Guarantor of the article:** Bharati Kochar, MD, MS, and Nneka N. Ufere, MD, MSCE.

**Specific author contributions:** B.K.: planning, drafting, and approval of final submitted version. N.N.U.: planning, drafting, and approval of final submitted version. C.S.R.: supervision, critical revision, and approval of final submitted version. J.C.L.: supervision, planning, critical revision, and approval of final submitted version.

**Financial support:** Funding for this study was supported in part by a Crohn's and Colitis Foundation Career Development Award (568735 to B.K.), American Association for the Study of Liver Diseases Advanced Transplant Hepatology Award (N.N.U.) and the National Institutes of Aging (R01AG059183 and R21AG067554 to J.C.L.).

**Potential competing interests:** B.K.: advisory board for Pfizer. N.N.U., C.S.R., and J.C.L.: no relevant conflicts of interest.

## References

[1] United States Census Bureau. 65 and Older Population Grows Rapidly as Baby Boomers Age. Updated October 8, 2021. (https://www.census.gov/newsroom/press-releases/2020/65-older-population-grows.html). Accessed October 8, 2021.

[2] Iriondo J Jordan J. An Aging Nation: Projected Number of Children and Older Adults 2018. Updated October 8, 2018. (https://www.census.gov/library/visualizations/2018/comm/historic-first.html). Accessed October 8, 2021.

[3] Bell F Miller M. Life Tables for the United States Social Security Area 1900–2100. Actuarial Study No. 120. Social Security Administration: Woodlawn, MD, 2005.

[4] Olshansky SJ. From lifespan to healthspan. JAMA 2018;320:1323. 30242384 10.1001/jama.2018.12621

[5] Kochar B Ufere NN Nipp R . Video-based telehealth visits decrease with increasing age. Am J Gastroenterol 2021;116:431–2. 33009048 10.14309/ajg.0000000000000961 PMC7553025

[6] Siegel RL Miller KD Fedewa SA . Colorectal cancer statistics, 2017. CA Cancer J Clin 2017;67:177–93. 28248415 10.3322/caac.21395

[7] Lucak S Lunsford TN Harris LA. Evaluation and treatment of constipation in the geriatric population. Clin Geriatr Med 2021;37:85–102. 33213776 10.1016/j.cger.2020.08.007

[8] John BK Bullock M Brenner L . Nutrition in the elderly. Frequently asked questions. Am J Gastroenterol 2013;108:1252–66; quiz 1267. 23711624 10.1038/ajg.2013.125

[9] Otaki F Iyer PG. Gastroesophageal reflux disease and Barrett esophagus in the elderly. Clin Geriatr Med 2021;37:17–29. 33213770 10.1016/j.cger.2020.08.003

[10] Chalasani N Fontana RJ Bonkovsky HL . Causes, clinical features, and outcomes from a prospective study of drug-induced liver injury in the United States. Gastroenterology 2008;135:1924–34.e4. 18955056 10.1053/j.gastro.2008.09.011 PMC3654244

[11] Dahlhamer J Zammitti E Ward B . Prevalence of inflammatory bowel disease among adults aged ≥18 years - United States, 2015. MMWR Morb Mortal Wkly Rep 2016;65:1166–9. 27787492 10.15585/mmwr.mm6542a3

[12] Coward S Clement F Benchimol EI . Past and future burden of inflammatory bowel diseases based on modeling of population-based data. Gastroenterology 2019;156:1345–53.e4. 30639677 10.1053/j.gastro.2019.01.002

[13] Haugen CE Holscher CM Garonzik-Wang J . National trends in liver transplantation in older adults. J Am Geriatr Soc 2018;66:2321–6. 30325004 10.1111/jgs.15583 PMC6289760

[14] Su F Yu L Berry K . Aging of liver transplant registrants and recipients: Trends and impact on waitlist outcomes, post-transplantation outcomes, and transplant-related survival benefit. Gastroenterology 2016;150:441–53.e6; quiz e16. 26522262 10.1053/j.gastro.2015.10.043

[15] Asrani SK Hall L Hagan M . Trends in chronic liver disease-related hospitalizations: A population-based study. Am J Gastroenterol 2019;114:98–106. 30333543 10.1038/s41395-018-0365-4

[16] Morley JE. A brief history of geriatrics. J Gerontol A Biol Sci Med Sci 2004;59:1132–52. 15602058 10.1093/gerona/59.11.1132

[17] Flaherty E Bartels SJ. Addressing the community‐based geriatric healthcare workforce shortage by leveraging the potential of interprofessional teams. J Am Geriatr Soc 2019;67:S400–S408. 31074849 10.1111/jgs.15924

[18] Kantor ED Rehm CD Haas JS . Trends in prescription drug use among adults in the United States from 1999–2012. JAMA 2015;314:1818–31. 26529160 10.1001/jama.2015.13766 PMC4752169

[19] Mehta RS Kochar BD Kennelty K . Emerging approaches to polypharmacy among older adults. Nat Aging 2021;1:347–356. 10.1038/s43587-021-00045-3 37117591

[20] By the 2019 American Geriatrics Society Beers Criteria Update Expert Panel. American Geriatrics Society 2019 updated AGS Beers Criteria for potentially inappropriate medication use in older adults. J Am Geriatr Soc 2019;67:674–94. 30693946 10.1111/jgs.15767

[21] O'Mahony D O'Sullivan D Byrne S . STOPP/START criteria for potentially inappropriate prescribing in older people: Version 2. Age and Ageing 2014;44:213–8. 25324330 10.1093/ageing/afu145 PMC4339726

[22] Scott IA Hilmer SN Reeve E . Reducing inappropriate polypharmacy. JAMA Intern Med 2015;175:827. 25798731 10.1001/jamainternmed.2015.0324

[23] Mate K Fulmer T Pelton L . Evidence for the 4Ms: Interactions and outcomes across the care continuum. J Aging Health 2021;2021:089826432199165. 10.1177/0898264321991658 PMC8236661 33555233

[24] Tinetti M Huang A Molnar F. The geriatrics 5M's: A new way of communicating what we do. J Am Geriatr Soc 2017;65:2115. 10.1111/jgs.14979 28586122

[25] King DE Xiang J Pilkerton CS. Multimorbidity trends in United States adults, 1988–2014. J Am Board Fam Med 2018;31:503–13. 29986975 10.3122/jabfm.2018.04.180008 PMC6368177

[26] Kochar B Kalasapudi L Ufere NN . Systematic review of inclusion and analysis of older adults in randomized controlled trials of medications used to treat inflammatory bowel diseases. Inflamm Bowel Dis 2021;27:1541–3. 33705536 10.1093/ibd/izab052

[27] Ananthakrishnan AN Donaldson T Lasch K . Management of inflammatory bowel disease in the elderly patient: Challenges and opportunities. Inflamm Bowel Dis 2017;23:882–93. 28375885 10.1097/MIB.0000000000001099 PMC5687915

[28] Alatab S Sepanlou SG Ikuta K . The global, regional, and national burden of inflammatory bowel disease in 195 countries and territories, 1990–2017: A systematic analysis for the Global Burden of Disease Study 2017. Lancet Gastroenterol Hepatol 2020;5:17–30. 31648971 10.1016/S2468-1253(19)30333-4 PMC7026709

[29] Bjarnason I Macpherson A Mackintosh C . Reduced bone density in patients with inflammatory bowel disease. Gut 1997;40:228–33. 9071937 10.1136/gut.40.2.228 PMC1027054

[30] Card T West J Hubbard R . Hip fractures in patients with inflammatory bowel disease and their relationship to corticosteroid use: A population based cohort study. Gut 2004;53:251–5. 14724159 10.1136/gut.2003.026799 PMC1774916

[31] Wang J Nakamura TI Tuskey AG . Polypharmacy is a risk factor for disease flare in adult patients with ulcerative colitis: A retrospective cohort study. Intest Res 2019;17:496–503. 31602961 10.5217/ir.2019.00050 PMC6821943

[32] Kochar B Cai W Cagan A . Frailty is independently associated with mortality in 11,001 patients with inflammatory bowel diseases. Aliment Pharmacol Ther 2020;52:311–8. 32537744 10.1111/apt.15821

[33] Tinsley A Navabi S Williams ED . Increased risk of influenza and influenza-related complications among 140,480 patients with inflammatory bowel disease. Inflamm Bowel Dis 2019;25:369–76. 30020478 10.1093/ibd/izy243

[34] Singh H Nugent Z Yu BN . Higher incidence of clostridium difficile infection among individuals with inflammatory bowel disease. Gastroenterology 2017;153:430–8.e2. 28479377 10.1053/j.gastro.2017.04.044

[35] Greuter T Vavricka S König AO . Malignancies in inflammatory bowel disease. Digestion 2020;2020:1–10. 10.1159/000509544 32799195

[36] Govani SM Wiitala WL Stidham RW . Age disparities in the use of steroid-sparing therapy for inflammatory bowel disease. Inflamm Bowel Dis 2016;22:1923–8. 27416039 10.1097/MIB.0000000000000817 PMC4956567

[37] Geisz M Ha C Kappelman MD . Medication utilization and the impact of continued corticosteroid use on patient-reported outcomes in older patients with inflammatory bowel disease. Inflamm Bowel Dis 2016;22:1435–41. 26978725 10.1097/MIB.0000000000000747 PMC4868778

[38] Rozich JJ Dulai PS Fumery M . Progression of elderly-onset inflammatory bowel diseases: A systematic review and meta-analysis of population-based cohort studies. Clin Gastroenterol Hepatol 2020;18:2437–47.e6. 32142940 10.1016/j.cgh.2020.02.048 PMC7490750

[39] Lobaton T Ferrante M Rutgeerts P . Efficacy and safety of anti-TNF therapy in elderly patients with inflammatory bowel disease. Aliment Pharmacol Ther 2015;42:441–51. 26104047 10.1111/apt.13294

[40] Cottone M Kohn A Daperno M . Advanced age is an independent risk factor for severe infections and mortality in patients given anti-tumor necrosis factor therapy for inflammatory bowel disease. Clin Gastroenterol Hepatol 2011;9:30–5. 20951835 10.1016/j.cgh.2010.09.026

[41] White MC Holman DM Boehm JE . Age and cancer risk. Am J Prevent Med 2014;46:S7–S15. 10.1016/j.amepre.2013.10.029 PMC4544764 24512933

[42] Zhavoronkov A. Geroprotective and senoremediative strategies to reduce the comorbidity, infection rates, severity, and lethality in gerophilic and gerolavic infections. Aging 2020;12:6492–510. 32229705 10.18632/aging.102988 PMC7202545

[43] Beaugerie L Brousse N Bouvier AM . Lymphoproliferative disorders in patients receiving thiopurines for inflammatory bowel disease: A prospective observational cohort study. Lancet 2009;374:1617–25. 19837455 10.1016/S0140-6736(09)61302-7

[44] Fox C Smith T Maidment I . Effect of medications with anti-cholinergic properties on cognitive function, delirium, physical function and mortality: A systematic review. Age Ageing 2014;43:604–15. 25038833 10.1093/ageing/afu096

[45] Fuller-Thomson E Sulman J. Depression and inflammatory bowel disease: Findings from two nationally representative Canadian surveys. Inflamm Bowel Dis 2006;12:697–707. 16917224 10.1097/00054725-200608000-00005

[46] Yesavage JA Sheikh JI. 9/Geriatric Depression Scale (GDS). Clin Gerontol 1986;5:165–73.

[47] Kochar B Barnes EL Long MD . Depression is associated with more aggressive inflammatory bowel disease. Am J Gastroenterol 2017;113:80–5. 29134965 10.1038/ajg.2017.423 PMC5962285

[48] Sachs-Ericsson N Joiner T Plant EA . The influence of depression on cognitive decline in community-dwelling elderly persons. Am J Geriatr Psychiatry 2005;13:402–8. 15879589 10.1176/appi.ajgp.13.5.402

[49] Zhang B Wang HE Bai YM . Inflammatory bowel disease is associated with higher dementia risk: A nationwide longitudinal study. Gut 2021;70:85–91. 32576641 10.1136/gutjnl-2020-320789

[50] Villumsen M Aznar S Pakkenberg B . Inflammatory bowel disease increases the risk of Parkinson's disease: A Danish nationwide cohort study 1977–2014. Gut 2019;68:18–24. 29785965 10.1136/gutjnl-2017-315666

[51] Kochar B Orkaby AR Ananthakrishnan AN . Frailty in inflammatory bowel diseases: An emerging concept. Therap Adv Gastroenterol 2021;14:175628482110254. 10.1177/17562848211025474 PMC8477705 34594400

[52] Page RL O'Bryant CL Cheng D . Drugs that may cause or exacerbate heart failure. Circulation 2016;134:e32–69. 27400984 10.1161/CIR.0000000000000426

[53] Ghosh S. Multidisciplinary teams as standard of care in inflammatory bowel disease. Can J Gastroenterol 2013;27:198. 23616956 10.1155/2013/710671 PMC3742475

[54] Nguyen GC Bernstein CN Benchimol EI. Risk of surgery and mortality in elderly-onset inflammatory bowel disease: A population-based cohort study. Inflamm Bowel Dis 2017;23:218–23. 27997435 10.1097/MIB.0000000000000993

[55] Schmidt ML Barritt AS Orman ES . Decreasing mortality among patients hospitalized with cirrhosis in the United States from 2002 through 2010. Gastroenterology 2015;148:967–77.e2. 25623044 10.1053/j.gastro.2015.01.032 PMC4430328

[56] Kwong AJ Devuni D Wang C . Outcomes of liver transplantation among older recipients with nonalcoholic steatohepatitis in a large multicenter US cohort: The re-evaluating age limits in transplantation consortium. Liver Transpl 2020;26:1492–503. 33047893 10.1002/lt.25863 PMC7960487

[57] Volk ML Tocco RS Bazick J . Hospital readmissions among patients with decompensated cirrhosis. Am J Gastroenterol 2012;107:247–52. 21931378 10.1038/ajg.2011.314 PMC3470789

[58] Hayward KL Weersink RA. Improving medication-related outcomes in chronic liver disease. Hepatol Commun 2020;4:1562–77. 33163829 10.1002/hep4.1612 PMC7603526

[59] Steinman MA Zullo AR Lee Y . Association of β-blockers with functional outcomes, death, and rehospitalization in older nursing home residents after acute myocardial infarction. JAMA Intern Med 2017;177:254–62. 27942713 10.1001/jamainternmed.2016.7701 PMC5318299

[60] Stahlmann R Lode HM. Risks associated with the therapeutic use of fluoroquinolones. Expert Opin Drug Saf 2013;12:497–505. 23651367 10.1517/14740338.2013.796362

[61] Hayward KL Horsfall LU Ruffin BJ . Optimising care of patients with chronic disease: Patient-oriented education may improve disease knowledge and self-management. Intern Med J 2017;47:952–5. 28782215 10.1111/imj.13505

[62] D'Amico G Garcia-Tsao G Pagliaro L. Natural history and prognostic indicators of survival in cirrhosis: A systematic review of 118 studies. J Hepatol 2006;44:217–31. 16298014 10.1016/j.jhep.2005.10.013

[63] Bajaj JS Wade JB Gibson DP . The multi-dimensional burden of cirrhosis and hepatic encephalopathy on patients and caregivers. Am J Gastroenterol 2011;106:1646–53. 21556040 10.1038/ajg.2011.157 PMC3989143

[64] Tapper EB Aberasturi D Zhao Z . Outcomes after hepatic encephalopathy in population-based cohorts of patients with cirrhosis. Aliment Pharmacol Ther 2020;51:1397–405. 32363684 10.1111/apt.15749 PMC7266029

[65] Bajaj JS Duarte-Rojo A Xie JJ . Minimal hepatic encephalopathy and mild cognitive impairment worsen quality of life in elderly patients with cirrhosis. Clin Gastroenterol Hepatol 2020;18:3008–16.e2. 32205222 10.1016/j.cgh.2020.03.033 PMC7502426

[66] Montagnese S Amodio P. Hepatic encephalopathy diagnosis conundrums. In: Bajaj JS (ed). Diagnosis and Management of Hepatic Encephalopathy: A Case-Based Guide. Springer International Publishing: Cham, Switzerland, 2018, pp 117–28.

[67] Rakoski MO McCammon RJ Piette JD . Burden of cirrhosis on older Americans and their families: Analysis of the health and retirement study. Hepatology 2012;55:184–91. 21858847 10.1002/hep.24616 PMC3462487

[68] Ezaz G Murphy SL Mellinger J . Increased morbidity and mortality associated with falls among patients with cirrhosis. Am J Med 2018;131:645–50.e2. 29453941 10.1016/j.amjmed.2018.01.026

[69] Tapper EB Nikirk S Parikh N . Falls are common, morbid, and predictable in patients with cirrhosis. J Hepatol 2021;75:582–8. 33887359 10.1016/j.jhep.2021.04.012 PMC8380639

[70] Lai JC Rahimi RS Verna EC . Frailty associated with waitlist mortality independent of ascites and hepatic encephalopathy in a multicenter study. Gastroenterology 2019;156:1675–82. 30668935 10.1053/j.gastro.2019.01.028 PMC6475483

[71] Lai JC Feng S Terrault NA . Frailty predicts waitlist mortality in liver transplant candidates. Am J Transplant 2014;14:1870–9. 24935609 10.1111/ajt.12762 PMC4107151

[72] Lai JC Covinsky KE Dodge JL . Development of a novel frailty index to predict mortality in patients with end-stage liver disease. Hepatology 2017;66:564–74. 28422306 10.1002/hep.29219 PMC5519430

[73] Wang S Whitlock R Xu C . Frailty is associated with increased risk of cirrhosis disease progression and death. Hepatology 2021. 10.1002/hep.32157 34528267

[74] Lai JC Sonnenday CJ Tapper EB . Frailty in liver transplantation: An expert opinion statement from the American Society of Transplantation Liver and Intestinal Community of Practice. Am J Transplant 2019;19:1896–906. 30980701 10.1111/ajt.15392 PMC6814290

[75] Targher G Byrne CD Lonardo A . Non-alcoholic fatty liver disease and risk of incident cardiovascular disease: A meta-analysis. J Hepatol 2016;65:589–600. 27212244 10.1016/j.jhep.2016.05.013

[76] Musso G Gambino R Tabibian JH . Association of non-alcoholic fatty liver disease with chronic kidney disease: A systematic review and meta-analysis. PLoS Med 2014;11:e1001680. 25050550 10.1371/journal.pmed.1001680 PMC4106719

[77] American Geriatrics Society Expert Panel on the Care of Older Adults with Multimorbidity. Patient-centered care for older adults with multiple chronic conditions: A stepwise approach from the American Geriatrics Society: American Geriatrics Society Expert Panel on the Care of Older Adults with Multimorbidity. J Am Geriatr Soc 2012;60:1957–68. 22994844 10.1111/j.1532-5415.2012.04187.x PMC4459791

[78] Kelly EM James PD Murthy S . Health care utilization and costs for patients with end-stage liver disease are significantly higher at the end of life compared to those of other decedents. Clin Gastroenterol Hepatol 2019;17:2339–46.e1. 30743007 10.1016/j.cgh.2019.01.046

[79] Ufere NN Donlan J Waldman L . Barriers to use of palliative care and advance care planning discussions for patients with end-stage liver disease. Clin Gastroenterol Hepatol 2019;17:2592–9. 30885884 10.1016/j.cgh.2019.03.022 PMC6745282

[80] Ufere NN Halford JL Caldwell J . Health care utilization and end-of-life care outcomes for patients with decompensated cirrhosis based on transplant candidacy. J Pain Symptom Manage 2020;59:590–8. 31655192 10.1016/j.jpainsymman.2019.10.016 PMC7024665

[81] Sudore RL Lum HD You JJ . Defining advance care planning for adults: A consensus definition from a multidisciplinary Delphi panel. J Pain Symptom Manage 2017;53:821–32.e1. 28062339 10.1016/j.jpainsymman.2016.12.331 PMC5728651

[82] Ufere NN Lai JC. Advance care planning in liver transplant-preparing for both life and death. JAMA Intern Med 2021;181:660–1. 33720282 10.1001/jamainternmed.2021.0149 PMC9576119

[83] Hurria A Levit LA Dale W . Improving the evidence base for treating older adults with cancer: American Society of Clinical Oncology statement. J Clin Oncol 2015;33:3826–33. 26195697 10.1200/JCO.2015.63.0319

[84] Alexander KP Newby LK Cannon CP . Acute coronary care in the elderly, part I: Non-ST-segment-elevation acute coronary syndromes: A scientific statement for healthcare professionals from the American Heart Association Council on Clinical Cardiology: In collaboration with the Society of Geriatric Cardiology. Circulation 2007;115:2549–69. 17502590 10.1161/CIRCULATIONAHA.107.182615

